# Flatfoot in Müller-Weiss syndrome: a case series

**DOI:** 10.1186/1752-1947-6-228

**Published:** 2012-08-01

**Authors:** Xu Wang, Xin Ma, Chao Zhang, Jia-Zhang Huang, Jian-Yuan Jiang

**Affiliations:** 1 Department of Orthopedics, Huashan Hospital, Fudan University, No. 12 Wulumuqi Middle Road, Shanghai, 200040, China

**Keywords:** Flatfoot, Müller-Weiss syndrome, Navicular

## Abstract

**Introduction:**

Spontaneous osteonecrosis of the navicular bone in adults is a rare entity, known as Müller-Weiss syndrome. We report here on our experience with six patients with Müller-Weiss syndrome accompanied by flatfoot deformity, but on a literature search found no reports on this phenomenon. Because the natural history and treatment are controversial, an understanding of how to manage this deformity may be helpful for surgeons when choosing the most appropriate operative procedure.

**Case presentation:**

Six patients (five women, one man; average age, 54 years) with flatfoot caused by osteonecrosis of the navicular bone were followed up between January 2005 and December 2008 (mean follow-up period, 23.2 months). Conservative treatment, such as physical therapy, and non-steroidal anti-inflammatory drugs were used, but failed. Physical examinations revealed flattening of the medial arch of the involved foot and mild tenderness at the mid-tarsal joint. Weight-bearing X-rays (anterior-posterior and lateral views), computed tomography, and MRI scans were performed for each case. Talonavicular joint arthrodesis was performed in cases of single talonavicular joint arthritis. Triple arthrodesis was performed in cases of triple joint arthritis to reconstruct the medial arch. Clinical outcomes were assessed using the American Orthopaedic Foot and Ankle Society ankle-hindfoot scale; the scores were 63.0 pre-operatively and 89.8 post-operatively. All patients developed bony fusion.

**Conclusions:**

The reason for the development of flatfoot in patients with Müller-Weiss syndrome is unknown. Surgical treatment may achieve favorable outcomes in terms of deformity correction, pain relief, and functional restoration. The choice of operative procedure may differ in patients with both flatfoot and posterior tibial tendon dysfunction.

## Introduction

Flatfoot is marked by reduction or collapse of the longitudinal arch. There are numerous causes of adult acquired flatfoot, including fracture, dislocation, tendon laceration, tarsal coalition, arthritis, neuroarthropathy, neurologic weakness, and iatrogenic causes. The development of flatfoot may result in pain and weakness of the entire lower extremity, walking difficulty, and other clinical symptoms. As a rare disease, osteonecrosis of the navicular bone may also lead to flatfoot, which is known as Müller-Weiss syndrome. The goal of flatfoot treatment is to eliminate the clinical symptoms and correct the malformation [1]. However, Müller-Weiss syndrome is a very rare disease, and there are few studies of this disease in terms of open surgical treatment in more than one patient. We adopted open operative methods to manage six patients with Müller-Weiss syndrome, and all subsequently achieved bony fusion and a pain-free condition.

## Case presentation

### Case 1

**Figure 1:**
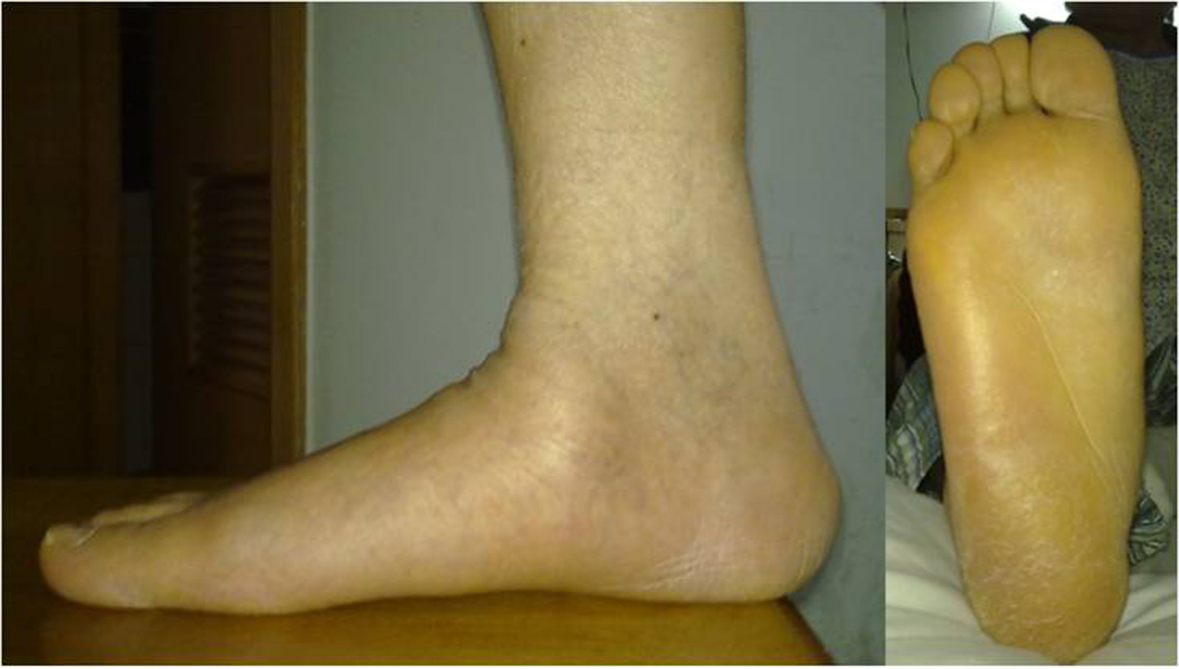
Flatting of the medial arch, neutral position of the hindfoot.

**Figure 2:**
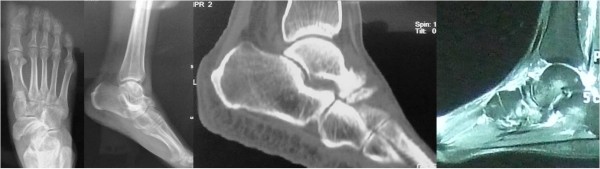
Weight-bearing X-rays, computed tomography (CT) and MRI scans from case 1.

**Figure 3:**
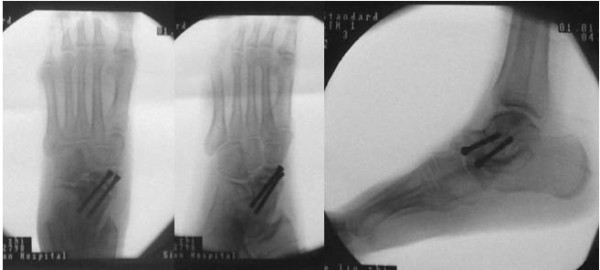
Post-operative X-ray of the talonavicular joint.

A 57-year-old Chinese woman presented to our hospital for evaluation of an approximately seven-year history of pain around the right ankle joint and medial side of the middle foot. The pain worsened during ambulation. A physical examination revealed flattening of the medial arch of the right foot and mild tenderness at the talonavicular joint, but the hindfoot was in a neutral position (Figure 1). Our patient underwent weight-bearing X-rays (anterior-posterior and lateral views), computed tomography (CT), and MRI scans (Figure 2). Conservative treatment failed, so we performed autografting and talonavicular joint arthrosis with two compressive screws (Figure 3).

### Case 2

**Figure 4:**
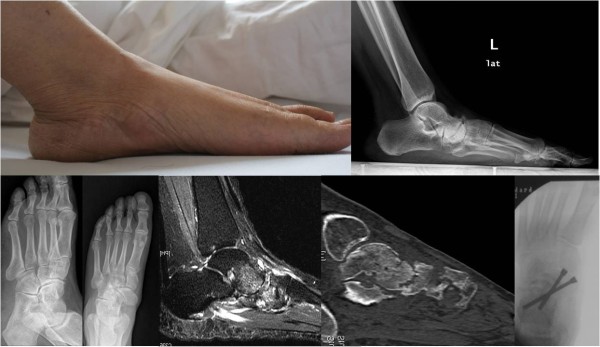
Case 2: bilateral Muller-Weiss syndrome, flatting of the medial arch, sinking of the talus head and arthrodesis of the talonavicular joint.

A 45-year-old Chinese woman presented to our hospital for evaluation of an approximately six-year history of pain around the bilateral ankle joints and medial side of the middle feet. The pain worsened during ambulation. A physical examination revealed flattening of the medial arch of both feet and mild tenderness at the talonavicular joint, but the hindfoot was in a neutral position. Our patient underwent weight-bearing X-rays (anterior-posterior and lateral views), CT, and MRI scans. Conservative treatment failed, so we performed autografting and talonavicular joint arthrosis with two compressive screws (Figure 4).

### Case 3

**Figure 5:**
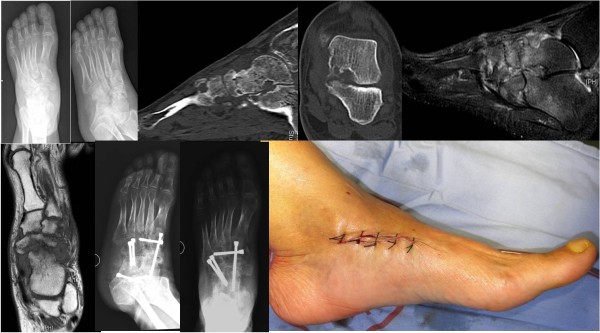
Case 3: Muller-Weiss Syndrome of the left foot, osteoarthritis of mid-tarsal joint and subtalar joint, triple arthrodesis and appearance operatively.

A 60-year-old Chinese woman presented to our hospital for evaluation of an approximately 10-year history of pain around the medial sides of the left middle feet. A physical examination revealed flattening of the medial arch of the left foot and mild tenderness at the mid-tarsal joint, but the hindfoot was in a neutral position. Our patient underwent weight-bearing X-rays (anterior-posterior and lateral views), CT, and MRI scans. Imaging data showed degenerative changes of the mid-tarsal and subtalar joints. Conservative treatment failed, so we performed triple arthrodesis and reconstructed the normal alignment of the hindfoot and middle foot (Figure 5).

### Case 4

**Figure 6:**
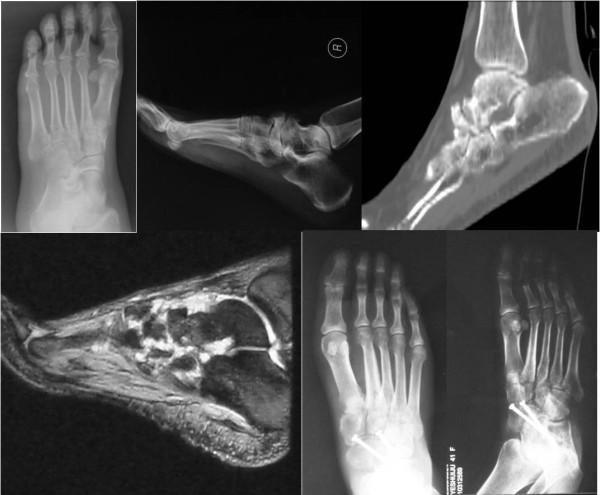
Case 4: Muller-Weiss syndrome of the right foot, osteoarthritis of talonavicular joint, talonavicular arthrodesis with two screws.

A 60-year-old Chinese woman presented to our hospital for evaluation of an approximately seven-year history of pain at the medial part of the right talonavicular joint during ambulation. A weight-bearing X-ray, CT, and MRI demonstrated navicular deformation. Arthrodesis of the talonavicular joint was performed (Figure 6).

### Case 5

**Figure 7:**
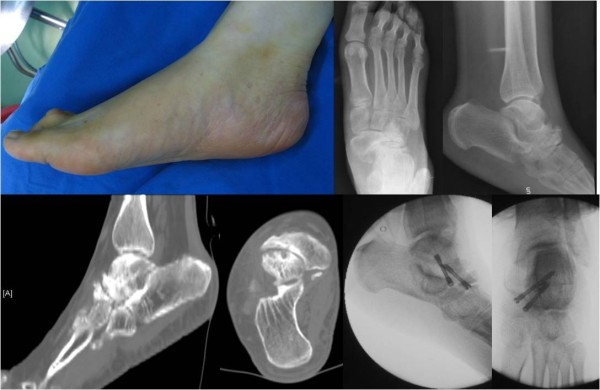
Case 5: flatfoot with Muller-Weiss syndrome of the right foot, osteoarthritis of talonavicular joint, talonavicular arthrodesis with two screws.

A 53-year-old Chinese woman presented to our hospital for evaluation of an approximately seven-year history of pain at the medial part of the right talonavicular joint during ambulation. A physical examination revealed flattening of the medial arch of the right foot. A weight-bearing X-ray and CT scan demonstrated navicular deformation and osteoarthritis of the talonavicular joint. Arthrodesis of the talonavicular joint was performed (Figure 7).

### Case 6

**Figure 8:**
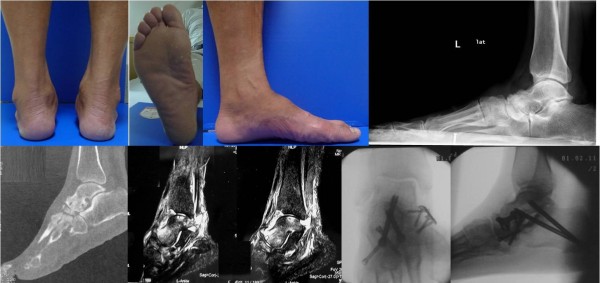
Case 6: flatfoot with Müller-Weiss syndrome of the left foot, the calcaneus varus and medial arch sunk, osteoarthritis of triple joints, triple arthrodesis with plates and screws.

A 49-year-old Chinese man presented to our hospital for evaluation of an approximately three-year history of pain around the left ankle during ambulation. A physical examination revealed flattening of the medial arch of the left foot with hindfoot varus. Imaging data showed triple osteoarthritis. Triple arthrodesis was performed to reconstruct the medial arch (Figure 8). The clinical appearance and imaging results revealed plantar displacement of the talus head and sinking of the medial arch of the foot. However, the hindfoot did not have valgus deformity and was almost in the neutral position. This phenomenon differs from the flatfoot that results from posterior tibial tendon dysfunction (PTTD).

The details of all our cases are listed in Table 1.

**Table 1:** **Patient data: demographics, disease characteristics, American Orthopaedic Foot and Ankle Society (AOFAS) scores**[2]**and time of follow-up**

**Case**	**Gender**	**Age**	**Duration, years**	**Stage**	**Foot affected**	**Surgery**	**Follow-up, months**	**Pre-operative AOFAS score**	**Post-operative AOPFAS score**	**Time in cast weeks**
1	Female	57	7	IV	Right	Talonavicular arthrodesis plus autologous iliac grafting	19	73	92	9
2	Female	45	6	IV	Bilateral	Talonavicular arthrodesis plus autologous iliac grafting	26	66	90	8
3	Female	60	10	V	Left	Triple arthrodesis	21	51	85	10
4	Female	60	7	V	Right	Talonavicular arthrodesis plus autologous iliac grafting	29	70	94	11
5	Female	53	7	IV	Right	Talonavicular arthrodesis plus autologous iliac grafting	17	61	88	9
6	Male	49	3	IV	Left	Triple arthrodesis	27	57	90	9

## Discussion

Spontaneous osteonecrosis of the tarsal navicular bone in adults, termed Müller-Weiss syndrome, differs from the well-recognized osteochondrosis of the tarsal navicular bone that occurs in children, termed Köhler disease. It was reported that multiple factors, such as damage to the blood supply of the tarsal navicular bone, result in abnormal ossification (chondrification and ossification) or osteocyte death in cartilage [3].

In the present series, early diagnosis of Müller-Weiss syndrome was difficult because of the delitescence of early symptoms, the vagueness of clinical characteristics, and the lower sensitivity of imaging tests. The imaging characteristics of osteonecrosis of the navicular bone are medial and/or dorsal protrusion with lateral collapse. The early imaging results showed that the lateral portion of the bone diminished and the translucency increased, followed by a comma-shaped deformity and protrusion and collapse of the dorsal portion [4]. CT defined the fracture line, and MRI scans showed a homogeneous decrease in the intensity on T1-weighted images. Thus, the auxiliary examination results were rather obvious when the chief complaint was intense pain.

All our patients suffered ankle and medial foot pain during walking, and the medial portion of the foot flattened with difficulty wearing shoes. Physical examination results showed collapse of the medial longitudinal arch and tenderness of the navicular bone and tarsal sinus. Some of our patients experienced tenderness along the posterior tibial muscle tendon. Both the change in talonavicular arthritis and the formation of osteophytes were significant on CT and MRI, indicating later-stage Müller-Weiss syndrome. The semi-developed lateral portion, which was secondary to blood supply shortage, mismatched the talus joint after the development of osteonecrosis of the navicular bone. The tarsal navicular bone gradually shifted dorsomedially in a repeated movement. Inflammatory changes in talonavicular articulation were found to be accompanied by the formation of a bone cyst under the talus and the intensity change [4].

Adult flatfoot is often clinically induced by PTTD. Thus, it was important to differentiate flatfoot in Müller-Weiss syndrome from PTTD. Generally, patients with PPTD in different stages showed various symptoms such as collapse of the medial longitudinal arch, forefoot abductus with supination, and hindfoot valgus deformity. The pain was concentrated at the ankle joint and tarsal sinus region. Our patients complained of significant foot deformity or problems wearing shoes. However, flatfoot caused by osteonecrosis of the navicular bone has no specific early symptoms or obvious radiographic evidence, which often leads to delayed diagnosis. The tarsal navicular bone developed deformities, fragmentation in the severe stages of the disease, and occasional involvement of the talonavicular articulation. The relationship between the forefoot and hindfoot was unusual in that the forefoot was located in the middle line of the ankle, the medial arch sunk, and the subtalar joint showed varus deformity. This phenomenon is contrary to the twist mechanics of the foot.

If conservative treatment fails to relieve the symptoms, surgical treatment should be proposed. The surgical technique aims to alleviate pain and maintain normal paratalar joint function. Although talonavicular arthrodesis is not recommended for treatment of flatfoot secondary to PTTD, it may be effective and efficient for isolated talonavicular osteoarthritis [5-7]. Considering the unusual dissection feature of the talonavicular articulation and the importance of the medial longitudinal arch, we decided to perform autologous iliac grafting and firm fixation to avoid an excessive osteotomy that may induce forefoot adduction deformity. Triple arthrodesis was performed in our patients with triple osteoarthritis. Patients should be well informed of the possibility of post-operative hindfoot stiffness [8].

## Conclusions

Müller-Weiss syndrome is rare and delitescent, but it greatly influences the foot function. It is necessary to diagnose and treat this condition early. Patients undergoing talonavicular arthrodesis should be followed up closely to prevent degeneration of adjacent joints [9]. Because the number of cases is small, we cannot compare the results of different operative procedures. We suggest that surgeons be aware of flatfoot in Müller-Weiss syndrome.

## Consent

Written informed consent was obtained from all patients for publication of this case report and any accompanying images. Copies of the written consents are available for review by the Editor-in-Chief of this journal.

## Competing interests

The authors declare that they have no competing interests.

## Authors’ contributions

JJ provided constructive comments. WX and MX performed the clinical work. ZC and HJ performed data collection and analysis. All authors read and approved the final manuscript.
